# Clinical Outcome of Patients Submitted to Liver Resection in the Context of Metastatic Breast Cancer: A Study of a Tertiary Hospital Center

**DOI:** 10.3390/medicines8110061

**Published:** 2021-10-20

**Authors:** Jorge Nogueiro, Vitor Devezas, Fabiana Sousa, Cristina Fernandes, Fernando Osório, Susy Costa, André Magalhães, Henrique Mora, Diana Gonçalves, Hugo Santos-Sousa, André Costa Pinho, Luís Graça, José Luís Fougo, Elisabete Barbosa

**Affiliations:** General Surgery, Centro Hospitalar Universitário São João, 4200-319 Porto, Portugal; vitor.devezas7@gmail.com (V.D.); fabiana.s.sousa52@gmail.com (F.S.); cris87ster@gmail.com (C.F.); cunha.osorio@gmail.com (F.O.); susycosta@sapo.pt (S.C.); amag1976@gmail.com (A.M.); henriquemmora@gmail.com (H.M.); dianafslg@hotmail.com (D.G.); h.santos.sousa@gmail.com (H.S.-S.); andrecostapinho@gmail.com (A.C.P.); luisafonsograca@gmail.com (L.G.); joseluisfougo@gmail.com (J.L.F.); elisabete_barbosa17@hotmail.com (E.B.)

**Keywords:** breast cancer, liver metastasis, prognosis, survival

## Abstract

Introduction: Breast cancer is the most incident cancer in the world, accounting for 25% of new cancers per year in females. It is the most frequent malignancy in women, being the fifth cause of death from cancer worldwide. Approximately 5 to 10% of patients already present with metastases at diagnosis, and the liver is the site of metastases in half of these cases. Liver metastasis (LM) resection, performed after neoadjuvant systemic treatment, has been reported to increase median overall survival in this population. Aim: The aim of this analysis is to assess the outcomes of patients undergoing breast cancer liver metastasis surgical resection, including impact on survival, compared to patients where metastasectomy was not performed. Methods: retrospective review of 55 female patients with breast cancer liver metastases, diagnosed and treated in a single tertiary university hospital from January 2011 to December 2016 was performed. Results: In 32/55 patients (58.2%), multi-organ metastases were identified (the most common sites being bone, lungs, and lymph nodes). Of the remaining 23 patients, the liver was the unique metastatic site; thirteen patients had diffuse bilobar hepatic metastases. The remaining ten patients were proposed for surgical treatment; three of them had peritoneal carcinomatosis identified during surgery, and no hepatic metastasectomy was performed. As a result, only seven (12.7%) patients underwent liver metastasectomy. Overall survival was higher in patients who had LM surgery (65 months [Interquartile Range (IQR) 54–120]), in comparison to those diagnosed with diffuse bilobar hepatic metastases (17.5 months [IQR 11–41]), and with those showing concurrent liver and bone metastases (16.5 months [IQR 6–36]) (*p* = 0.012). In univariable analysis, the latter two groups showed worse overall survival outcomes (Hazard Ratio (HR) = 3.447, 95%CI: 1.218–9.756, *p* = 0.02 and HR = 3.855, 95% Confidence Interval (CI): 1.475–10.077, *p* = 0.006, respectively) when compared to patients with LM. Conclusion: In our series, patients submitted to metastasectomy had a median overall survival after diagnosis of LM three times greater than the non-operated patients with isolated LM, or concurrent LM and bone metastases (65 vs. 17.5 and 16.5 months, respectively). As is vastly known for colorectal cancer liver metastasis, resection of breast cancer liver metastasis may reduce tumor burden, and therefore may improve patient outcome.

## 1. Introduction

Breast cancer, with an incidence of 11.7% in both sexes in 2020, is the most incident cancer in the world, and the fifth cause of death from cancer worldwide [1,2].

Approximately 5 to 10% of the patients already present with metastases at diagnosis. Among patients with metastatic breast cancer, the liver is the site of metastases in half of cases, mostly in association with additional sites (bone, pleura/lung, central nervous system, etc.) [3]. In up to 10% of cases, breast cancer liver metastases (LM) may occur in an isolated form. LM have been generally considered as a disseminated disease with poor prognosis, and one of the events responsible for the increased number of deaths in women with breast cancer [4]. Treatment with systemic chemotherapy and/or endocrine therapy rarely results in a complete response, and overall survival after LM diagnosis ranges from 3 to 26 months [5].

With the benefits demonstrated after resection of LMs from colorectal cancer in centers with considerable experience in liver surgery, the enthusiasm for extending LM resections to other malignancies (namely, for breast cancer patients) has increased. Indeed, the first reported series of hepatectomies performed in the context of metastatic breast cancer was published in 1991 [6,7].

LM resection, performed after neoadjuvant systemic treatment, was reported in some studies to result in an increased median overall survival of up to 116 months, and a 5-year survival outcome of up to 78% [3,5,8,9,10].

Treatment of metastatic breast cancer has undergone considerable changes (in part due to improved molecular classification and refined biomarkers of the disease), and recent advances in systemic therapies have significantly increased survival. Regarding breast cancer LM, it is advocated that selected patients may benefit from combining such systemic therapies with an approach directed towards the liver disease, including performing surgical resection. However, more precise ways of identifying patients that would benefit the most from LM resection are needed, since patients with stage IV breast cancer are considered patients with very poor prognosis, and are rarely referred to surgical consultation.

The aim of this analysis is to assess the outcomes of patients undergoing breast cancer LM surgical resection, including the impact on survival outcomes, and contribute to clarify the role of hepatic resection in these patients.

## 2. Materials and Methods

This is a retrospective investigation of the clinical files of 55 female patients with breast cancer (median age: 48 years; range: 24–80 years), with histologically-proven breast cancer LMs, diagnosed and treated in a single tertiary university hospital from January 2011 to December 2016. Follow-up was last updated in March 2021, totalizing a median follow-up of 74 [IQR 47–125] months.

The following clinicopathological variables were collected after careful review of patient clinical files: age at diagnosis; primary tumor size and histology; perineural and lymphovascular invasion; Estrogen Receptor (ER), Progesterone Receptor (PR) and Human Epidermal growth factor Receptor 2 (HER2) status; primary treatment; type of resection; LM characteristics; LM resection; dates of diagnosis, surgery, LM and last follow-up. Some patients were primary treated in another institution, thus some clinicopathological data were not available.

Clinicopathological characteristics of the study cohort are summarized in Table 1.

Hormonal receptor status was considered positive if immunostaining was present in ≥1% of tumor cells. HER2 status was considered positive if 3+ had immunostaining present, or in the case of 2+, were followed by Fluorescence in situ hybridization (FISH) amplification. Molecular subtypes of breast cancer were assessed based on immunohistochemistry, as recommended.

Cumulative survival curves for overall survival (OS) were computed using the Kaplan–Meier (KM) method, and log rank test was used to assess differences between groups. Hazard ratio (HR) and respective 95% confidence intervals (CI) were calculated by using the Cox regression model.

Statistical analysis was performed using SPSS^®^ 26.0 for Mac (IBM Co., Armonk, NY, USA). Percentages were computed based on cases with available information.

Significance was assumed for *p*-values inferior to 0.05. All *p*-values given were results of two-sided tests.

## 3. Results

The median age of the patients was not significantly different among groups (48, 42.5, 49.5 years for resected LM, non-resected LM, and multi-organ metastases, respectively).

Regarding the primary tumor histology of the patients submitted to liver metastasectomy, 4 patients had an invasive ductal carcinoma, 1 patient had an invasive lobular carcinoma, and 2 patients had a mixed carcinoma (mucinous and ductal; lobular and ductal). Of note, 1 patient was diagnosed with ductal carcinoma in situ, and no microinvasion was reported at the time; however, the patient recurred with metastatic dissemination.

The vast majority of the cohort belonged to the luminal molecular subtype, and 6/7 of the patients submitted to LM resection had luminal tumors, with only one simultaneously having HER2 amplification.

Median overall survival after diagnosis of LM was of 20 [IQR 9–47] months. The median time elapsed between breast surgery and LM diagnosis was 47 (IQR range: 21–74) months (with three cases of synchronous LM).

**Figure 1:**
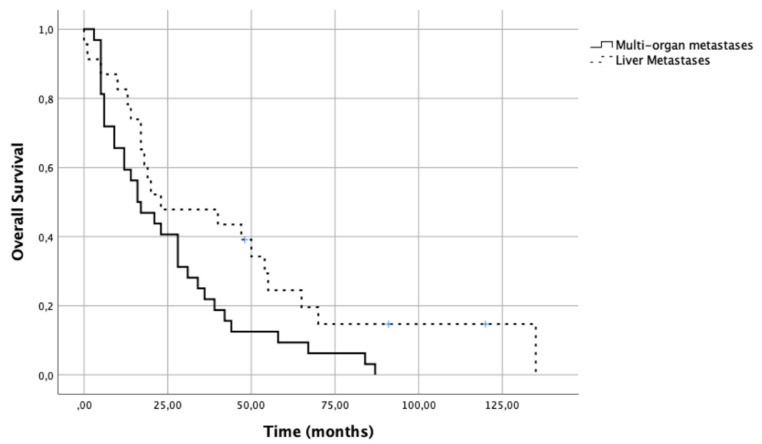
Survival analysis of breast cancer patients according to metastasis profile.

In 32/55 patients (58.2%), multi-organ metastases were identified (the most common sites being bone, lungs, and lymph nodes). Median survival after diagnosis of LM in these patients was 16.5 months [IQR 6–35.5]. Median survival after diagnosis of LM in patients with only liver metastases was 23.0 months [IQR 14–55] (Figure 1)

Of the remaining 23 patients, liver was the unique metastatic organ; 13 patients had diffuse bilobar hepatic metastases. The remaining 10 patients were proposed for surgical treatment; three of them had peritoneal carcinomatosis identified during surgery, and no hepatic metastasectomy was performed.

**Figure 2:**
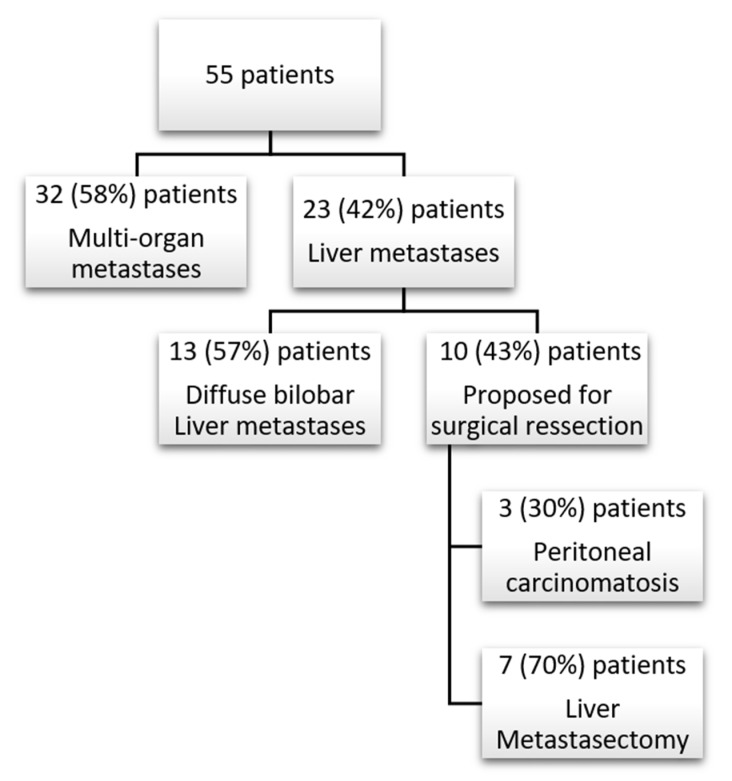
Study Flowchart.

As a result, only 7 (12.7%) patients underwent liver metastasectomy (Figure 2). The median time elapsed between breast surgery and LM diagnosis in these patients was 23 months. Of these, three required a re-metastasectomy, due to an in-liver recurrence. Median time elapsed between breast surgery and LM diagnosis in these patients was 23 months [IQR 1–28], and six of them received neoadjuvant systemic treatment with chemotherapy for LM. There was no mortality or major morbidity (Clavien–Dindo score ≥ 3) in these procedures.

**Figure 3:**
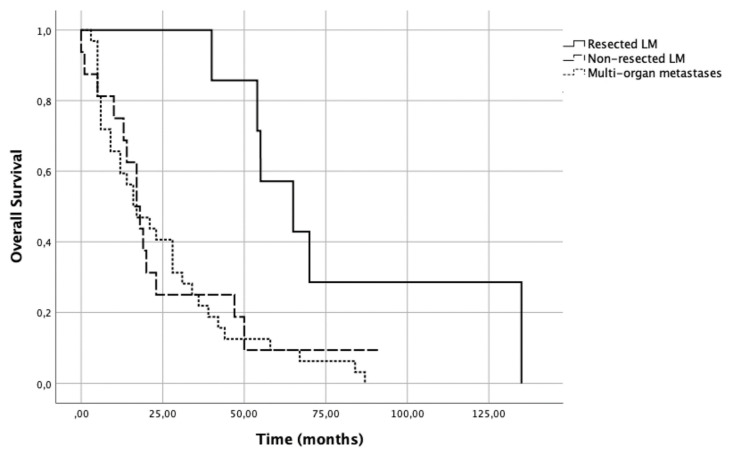
Survival analysis of breast cancer patients according to LM treatment.

Overall survival was significantly higher in patients who had LM surgery (65 months [IQR 54–120]), in comparison to those diagnosed with diffuse bilobar hepatic metastases (17.5 months [IQR 11–41]), and to those showing concurrent liver and extra-hepatic metastases (16.5 months [IQR 6–36]) (*p* = 0.012) (Figure 3). In univariable analysis, the latter two groups showed significantly worse overall survival outcomes (HR = 3.447, 95%CI: 1.218–9.756, *p* = 0.02 and HR = 3.855, 95%CI: 1.475–10.077, *p* = 0.006, respectively) when compared to patients submitted to LM.

## 4. Discussion

Metastatic breast cancer is generally believed to be associated with a poor prognosis. LM from breast cancer is not an infrequent form of systemic disease, due to the hematologic spread of tumor cells [4,8,9].

Systemic therapies should be considered for the majority of patients with LM, despite its known limitations. Other treatment modalities such as medical therapies or selective internal radiation therapy (SIRT) have been advocated to have similar results as surgical approaches in this population [11,12]. Even though the role of breast LM resection is not clarified, as there are not sufficient data to support it, patients with small oligometastases confined to the liver, diagnosed one year or more after treatment of the primary tumor, and responding to systemic therapy, should be evaluated in a multidisciplinary meeting for liver metastasectomy [13,14].

Golse and Adam (2017) described the most accurate patient-tumor framework to obtain the foremost outcomes after breast LM resection: small metastases (<4–5 cm), single or not, requiring major hepatectomy; radical resection (ideally R0, or R1 if necessary); stable disease (or ideally in regression) after neoadjuvant systemic treatment; and a delay between primary and secondary lesions longer than one-two years [9,15].

Neoadjuvant chemotherapy for breast cancer LM introduced an improvement in overall results; this is due to better systemic control, both to the reduction of the risk of in-liver recurrence, and to an increase in long-term survival outcomes [9].

In fact, liver resection for breast cancer LM is seldom referred for surgical evaluation, since stage IV breast cancer is generally perceived as a systemic disease with a very poor prognosis. However, with resections of breast cancer LM, in selected cases, some authors reported an increase in overall survival outcomes that may reach 116 months [3,5].

This way, as is already vastly known for colorectal cancer liver metastasis, resection of breast cancer liver metastasis may reduce tumor burden, and therefore may improve patient outcome.

In our series, patients submitted to metastasectomy had a median overall survival after diagnosis of LM three times greater than those patients with isolated LM or concurrent LM and bone metastases who were not submitted to surgery (65 vs. 17.5 and 16.5 months, respectively).

All published series conclude that randomized clinical trials are needed to definitively determine the advantage of metastasectomy in LM breast cancer. Management of these complex patients by an experienced multidisciplinary team (in highly specialized centers, with considerable expertise in liver surgery) is essential for providing the best treatment options, and achieve better results.

The role of liver resection in patients with extra-hepatic disease is still controversial in the literature. Patients with multi-organ metastasis have, by nature, a more aggressive cancer, and the prognosis of these group of patients may be worse than in patients with liver metastasis only. There are also few reports with no survival difference between patients with isolated liver metastasis and those with multi-organ metastasis. [10] In our cohort, patients with multi-organ metastases were not considered for any surgical resection after discussion in a multidisciplinary meeting, and, in our cohort, patients submitted to metastasectomy had a better survival. In this way, we aim to provide some more data to the discussion on which approach is best for these groups of patients, and to recall surgical approach as a viable option that needs to be discussed in multidisciplinary meetings, comparable to the surgical approach already considered for colorectal liver metastasis.

Our work has limitations, namely being a unicentric study, which may have led to bias in the study population selection. There is a bias in the patient cohort, since unifocal LM may already have a better outcome compared to those patients with multiple LM or disseminated disease. However, this study demonstrates that LM resection is an advantageous treatment option for breast cancer patients with isolated LM.

The study is also retrospective in nature, and has a limited number of patients. In the future, larger multicentric studies are required to better conclude the clinical benefit of LM resection in metastatic breast cancer patients.

## Figures and Tables

**Table 1:** Clinicopathological features of the study cohort.

	Resected LM	Non-Resected LM	Multi-Organ Metastases
Age (med, IQR)	48 (39–58)	42.5 (25–55)	49.5 (43.25–54.00)
Primary tumor size (med, IQR)	29 (19–30)	29.5 (25–31.25)	26 (14.5–40)
Primary tumor histology			
DCIS	0	0	1
IDC	4	12	22
ILC	1	1	4
Other	2	1	3
Pathological T stage (pT/ypT)			
Tis	0	1	2
1	3	3	8
2	4	9	15
3	0	2	2
4	0	0	1
Pathological N stage (pN/ypN)			
0	2	7	13
1	3	2	4
2	2	5	8
3	0	1	2
Margin Status (R)			
0	6	14	27
1	1	1	0
Lymph vessel invasion			
No	4	5	11
Yes	3	7	14
Blood vessel invasion			
No	5	10	22
Yes	2	2	3
Perineural invasion			
No	7	9	20
Yes	0	3	0
ER (≥1%)			
No	0	2	1
Yes	7	13	31
PR (≥1%)			
No	2	6	5
Yes	5	8	26
HER2			
Neg	6	13	30
Pos	1	2	2
Molecular subtype			
Luminal	6	11	29
Luminal-HER2	1	2	2
Triple negative	0	2	1
Primary treatment			
Surgery	6	8	21
Chemotherapy	1	8	11
Type of resection			
Tumorectomy	4	9	11
Mastectomy	3	7	19
Adjuvant Chemoterapy			
No	1	1	4
Yes	6	14	26
Hormone therapy			
No	0	2	2
Yes	7	13	29
Trastuzumab			
No	6	12	30
Yes	1	2	2
Radiotherapy			
No	0	2	8
Yes	6	14	23

Abbreviations: Liver Metastasis (LM); Interquartile Range (IQR); Ductal Carcinoma in situ (DCIS); Invasive Ductal Carcinoma (IDC); Invasive Lobular Carcinoma (ILC); Estrogen Receptor (ER), Progesterone Receptor (PR) and Human Epidermal growth factor Receptor 2 (HER2).
